# Critical illness-associated cerebral microbleeds involving the corpus callosum following cardiac arrest: A case report

**DOI:** 10.1097/MD.0000000000039273

**Published:** 2024-08-09

**Authors:** Bahadar S. Srichawla, Ton Fang, Vincent Kipkorir, Rakhee Lalla

**Affiliations:** a Department of Neurology, University of Massachusetts Chan Medical School, Worcester, MA; b Department of Medicine, University of Nairobi, Nairobi, Kenya.

**Keywords:** anoxic brain injury, cardiac arrest, cerebral microbleeds, CI-aCMBs, corpus callosum, critical illness associated cerebral microbleeds

## Abstract

**Rationale:**

Critical illness-associated cerebral microbleeds (CI-aCMBs) are emerging as significant radiographic findings in patients with hypoxic ischemic injuries. Their occurrence, particularly in the corpus callosum, warrants a closer examination due to the potential implications for neurological outcomes in critically ill patients. We aim to describe a rare case of CI-aCMBs within the corpus callosum following cardiac arrest with the goal of bolstering the scientific literature on this topic.

**Patient concerns:**

A 34-year-old man with a history of polysubstance abuse was found unconscious and experienced a pulseless electrical activity (PEA) cardiac arrest after a suspected drug overdose. Post-resuscitation, the patient exhibited severe respiratory distress, acute kidney injury, and profound neurological deficits.

**Diagnoses:**

Initial magnetic resonance imaging scans post-cardiac arrest showed no acute brain abnormalities. However, subsequent imaging revealed extensive cerebral microbleeds predominantly in the corpus callosum, diagnosed as CI-aCMBs. These findings were made in the absence of high signal intensity on T_2_-weighted images, suggesting a unique pathophysiological profile of microhemorrhages.

**Interventions:**

The patient underwent targeted temperature management (TTM) and supportive care in the intensive care unit after cardiac arrest.

**Outcomes:**

He was subsequently extubated and had significant recovery without any neurological deficits.

**Lessons:**

CI-aCMBs is a rare radiographic finding after cardiac arrest. These lesions may be confined to the corpus callosum and the long-term clinical and radiographic sequelae are still largely unknown.

## 1. Introduction

Cardiac arrest remains a critical medical emergency that necessitates immediate intervention to preserve life and minimize neurological deficits.^[1]^ Advances in resuscitation and post-resuscitation care have improved survival rates, but neurological sequelae among survivors continue to pose significant challenges for clinicians.^[2]^ One of the lesser known but complications following hypoxic-ischemic injury is the development of cerebral microbleeds (CMBs). These small focal areas of blood products within the brain parenchyma are often detected incidentally on magnetic resonance imaging (MRI) and have been associated with various pathophysiological mechanisms, including hypertension, amyloid angiopathy, and, particularly, hypoxia.^[3]^

Critical illness-associated cerebral microbleeds (CI-aCMBs) are a distinct clinical radiographic finding that involves the acute onset of CMBs often in critically ill patients in the intensive care unit (ICU). Reported etiologies include anoxic brain injury (ABI), extracorporeal membrane oxygenation (ECMO), acute respiratory distress syndrome (ARDS), high-altitude cerebral edema (HACE), and toxins, among others.^[4]^ The corpus callosum, a critical white matter structure facilitating interhemispheric communication, is seldom highlighted in the literature as a locus for CMBs despite its vulnerability to hypoxic-ischemic injuries.^[5]^ The presence of CMBs in this region could have profound implications for cognitive and functional recovery, yet it remains under investigation. This case report aims to better characterize the clinical and radiographic manifestations of CI-aCMB that preferentially involve the corpus callosum without abnormal high-signal intensity on T_2_-weighted images in a young patient after cardiac arrest. This case report was completed following the CARE guidelines.

## 2. Methods

**Figure 1.**
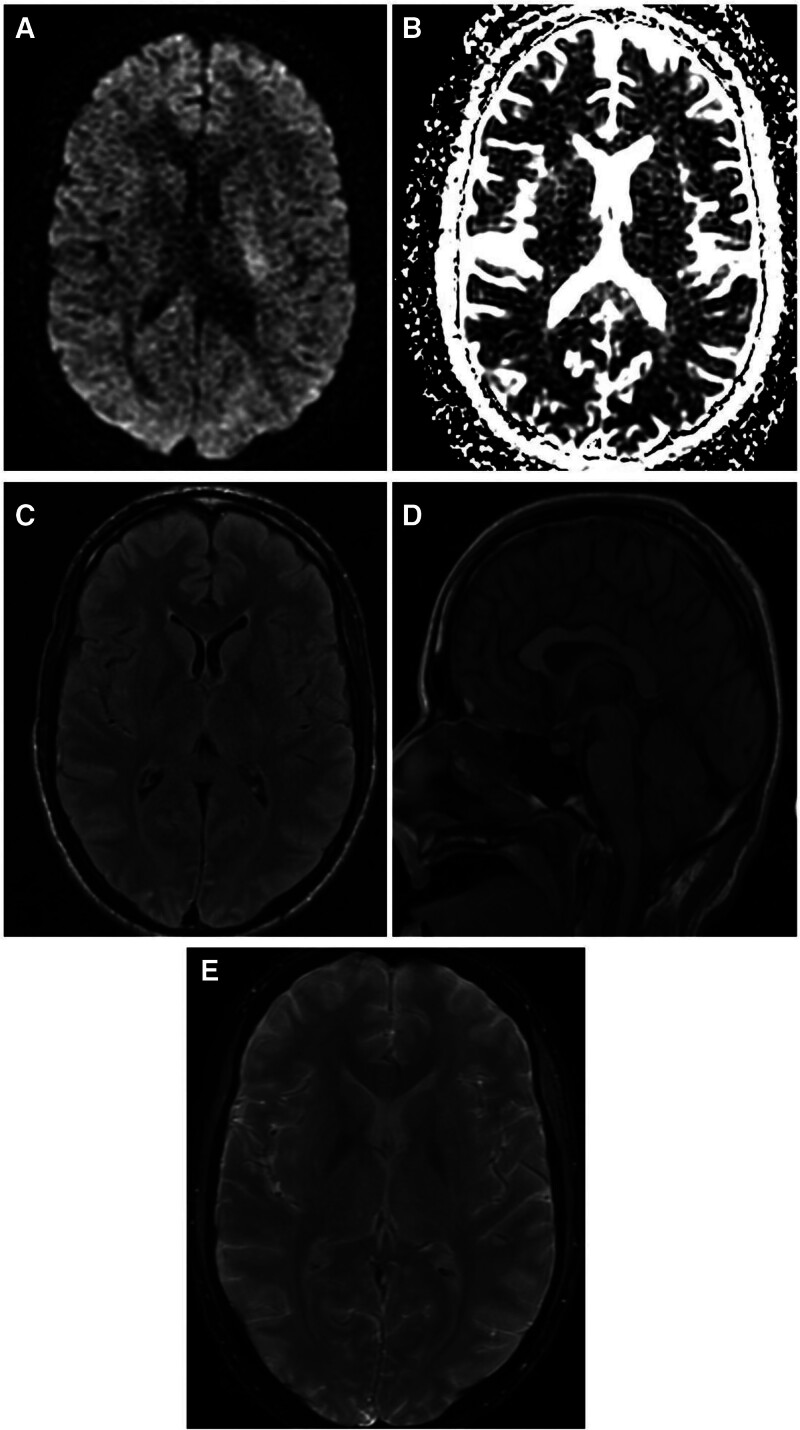
MRI of the brain (A) Diffusion weighted sequence and (B) apparent diffusion coefficient depicting T_2_ shine through artifact without true ischemic changes. (C) Axial T_2_-weighted FLAIR sequence and (D) coronal T_1_-weighted sequence. (E) Susceptibility weighted imaging sequence without hypointensities. FLAIR = fluid-attenuated inversion recovery, MRI = magnetic resonance imaging.

**Figure 2.**
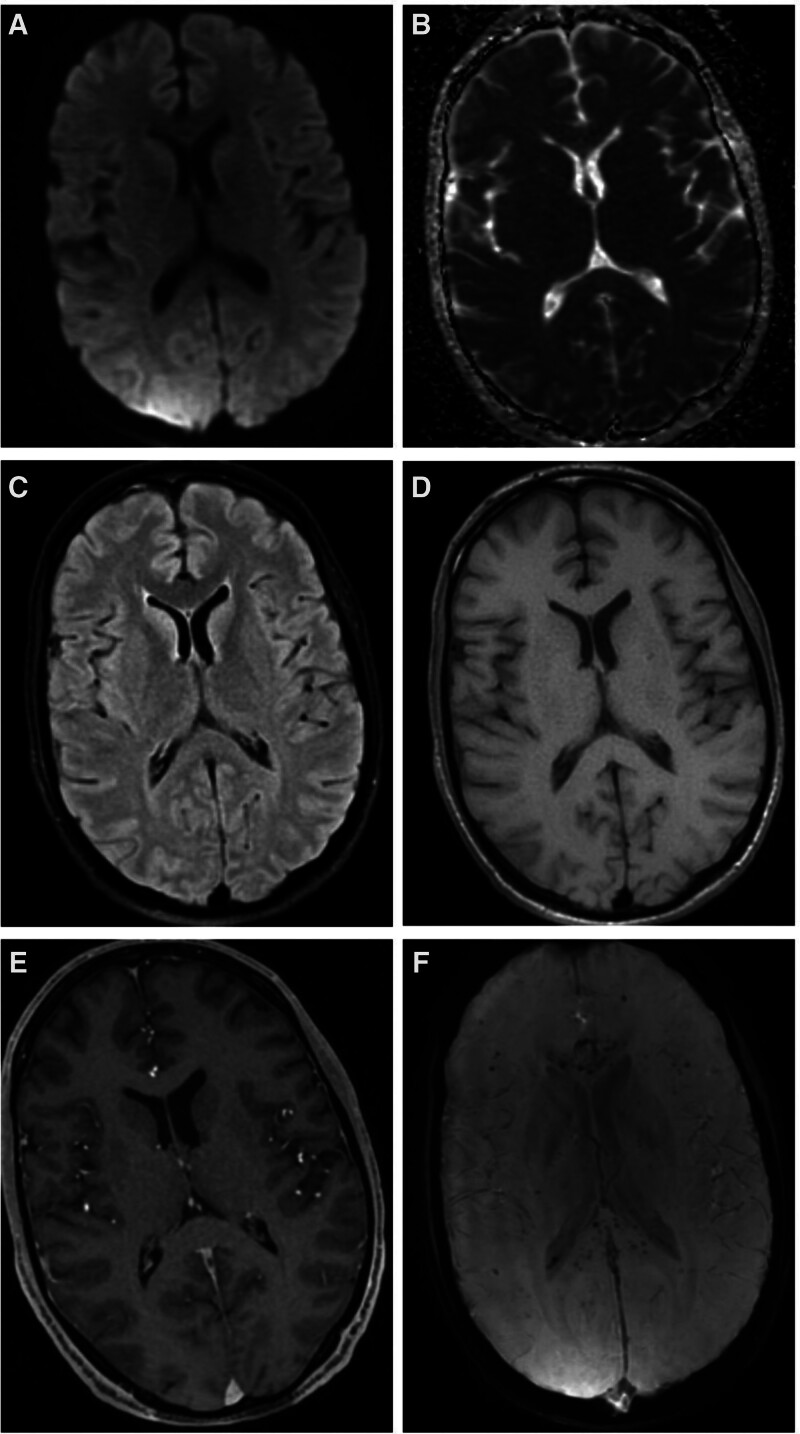
MRI of the brain (A) Diffusion weighted sequence and (B) apparent diffusion coefficient without any diffusion restriction. (C) Axial T_2_-weighted FLAIR sequence and (D) axial pre-contrast T_1_-weighted sequence. (E) Axial post-contrast T_1_-weighted sequence. (F) Susceptibility weighted imaging sequence showing juxtacortical hypointensities and significant hypointensities within the corpus callosum. FLAIR = fluid-attenuated inversion recovery, MRI = magnetic resonance imaging.

A 34-year-old man with a medical history of polysubstance abuse was found in his bathroom by a family member from suspected drug overdose. He was intubated in the field and brought to the emergency department with pulseless electrical activity (PEA) cardiac arrest and spontaneous return of circulation (ROSC) was obtained in 5 minutes. The initial physical examination shows a tachycardic male with no visible trauma. Diminished breath sounds in the right upper lobe with wheezing present. The Glasgow Coma Scale (GCS) was 3. The initial arterial blood gas was consistent with severe respiratory acidosis (pH 7.10, HCO3 24.7 mEq/L, PaCO2 80.3 mm Hg, PO2 116 mm Hg) and lactic acid 18.25 mmol/L. He started the targeted temperature management protocol (TTM) and his toxicology test was positive for opioids. A MRI of the brain was completed that showed no acute brain abnormalities (Fig. 1). His ICU course was further complicated by an acute kidney injury. He was subsequently extubated ICU-day 7. On hospital day 20 the patient developed worsening respiratory distress and was re-intubated and admitted to the ICU. The neurology team was consulted for the presence of weakness on physical examination. The neurological examination showed an intubated atraumatic male with a GCS of 5 (E_2_V_1_M_2_) and a FOUR score of 10 (E_4_M_1_B_4_R_1_). The patient had a lack of motor response to central and peripheral pain stimuli in all 4 extremities. An MRI of the cervical spine was completed and showed no acute abnormalities or spinal stenosis. An MRI of the brain with and without contrast did not show acute infarction or increased intensity on T_2_-weighted imaging. Susceptibility-weighted imaging showed extensive hypointensities consistent with microhemorrhages involving juxtacortical white matter in the bilateral cerebral hemispheres, as well as significant involvement of the corpus callosum (Fig. 2). A continuous electroencephalogram (EEG) was completed for 72 hours without showing seizure activity. The patient showed a significant improvement in his weakness and was subsequently extubated on day 17. Informed consent was obtained for the publication of this report.

## 3. Discussion

Our case of a young man found to have CI-aCMBs after a PEA cardiac arrest with significant corpus callosum involvement highlights the intriguing intersection between radiographic findings and pathophysiological underpinnings of microhemorrhages from ABI. Kim et al conducted a study to investigate the relationship between callosal microbleeds and ABI. The study involved 27 patients with ABIs, who were retrospectively compared to a control group without such injuries. The research found a significantly higher prevalence of CMBs in the patient group (29.6%) compared to the control group (3.7%), with all observed microbleeds located in the corpus callosum. Among callosal microbleed patients, there was a noted, albeit nonsignificant, trend toward a better prognosis, fewer typical magnetic resonance findings of ABI, and a higher incidence of cardiopulmonary resuscitation. Their study also showed a mean time of 8.41 days from ABI to the development of CMBs. A similar temporal profile to the patient that is reported here. These findings suggest that callosal microbleeds could serve as an adjunctive MRI marker to assess ABI, although the observed differences did not achieve statistical significance.^[6]^

Naim et al^[7]^ explored the occurrence of CMBs in severely poisoned patients with atypical neurological outcomes. Their retrospective study reviewed brain MRIs of patients admitted to an ICU from 2014 to 2021. Out of 2986 severely poisoned patients, only 3 (4.7%) of the 64 who underwent MRI presented with CMBs. These microbleeds were mainly located in the white matter of the corpus callosum. Patients, who had ingested toxic substances such as dichlorvos, methadone, and tramadol, experienced severe hypoxemia, required mechanical ventilation, and exhibited delayed arousal and dysexecutive syndromes that lead to long-term sequelae. The study suggests that CMBs in these patients are likely multifactorial and are not directly related to ingested toxicants.^[7]^ Topiwala et al conducted a retrospective analysis of 307 patients who received ECMO support at a tertiary care university hospital between 2013 and 2018 to investigate the prevalence, radiographic patterns, and clinical correlates of possibly-ECMO-related CMBs. Of the 40 patients who underwent an MRI within 3 months of ECMO support, 77.5% exhibited CMBs. A significant proportion of these patients (71%) displayed CMBs, predominantly affecting the splenium. The study categorized the CMBs into 3 levels of severity: low (<10 CMBs), moderate (10–30 CMBs), and high (>30 CMBs). Clinical outcomes revealed that patients with CMBs had higher incidences of ischemic stroke, intracranial hemorrhage, and all-cause mortality compared to those without CMBs. However, there was no significant correlation between leukoaraiosis and the presence or burden of CMBs.^[8]^

In addition to ABI, infections linked to the development of CMBs include SARS-CoV-2. A 2023 systematic review conducted aimed to characterize CMBs in critically ill patients, particularly comparing those associated with COVID-19 to other conditions. A literature search across PubMed and Embase yielded 23 relevant studies comprising 143 cases, predominantly male (73%) with a median age of 61. Many cases (73%) were linked to SARS-CoV-2-associated pneumonia. Key findings include a median ICU stay of 34 days and median mechanical ventilation duration of 24 days. Microbleeds were commonly located juxtacortical (79%), in the corpus callosum (75%), and deep white matter (71%), across both patient groups. Brainstem microbleeds were more frequent in patients without COVID-19. Furthermore, non-COVID-19 patients were generally younger and had significantly lower median platelet counts compared to those with COVID-19.^[9]^

The exact pathophysiological underpinnings of CI-aCMBs as well as preferential involvement of the corpus callosum are not well understood. Generally, the presence of CMBs without prominent vasogenic edema on T_2_-weighted imaging points towards microvascular/capillary endothelial failure leading to microhemorrhages seen on SWI. These atypical radiographic findings have also been reported in cases of HACE.^[10,11]^ Vessel anatomy may potentially play a role in the development of CMBs of the corpus callosum. The presence of short perforating arteries from the pericallosal artery supplying the corpus callosum may make it prone to autoregulatory failure.^[12]^ Additionally, the corpus callosum’s high energetic demand, decreased adrenergic tone may also be a contributing factor to the development of CMBs in this structure.^[13,14]^

The presence of CI-aCMBs, particularly in the corpus callosum after events like cardiac arrest, faces several challenges and holds multiple avenues for future research. The major limitation of this case study is being a single retrospective report. Future studies need to delve into the underlying mechanisms of CI-aCMBs, with a focus on microvascular changes and endothelial dysfunction. Longitudinal and comparative studies are essential to understand the progression and impact of CI-aCMBs on neurological functions and to compare these effects in different etiologies of brain injury. Furthermore, the exploration of therapeutic strategies to mitigate CI-aCMBs and the use of advanced imaging techniques can improve our understanding of their clinical importance.

## 4. Conclusions

This case study highlights the occurrence of CI-aCMBs with a notable involvement of the corpus callosum in a young patient following a cardiac arrest due to a suspected drug overdose. This case underscores the complexity of neurological manifestations that can arise after hypoxic-ischemic injuries and the potential role of CI-aCMBs as a diagnostic marker in the critical care setting. The findings suggest that the corpus callosum, despite its critical role in interhemispheric communication, remains a vulnerable target during ABI, possibly due to its unique vascular supply and high metabolic demands. The presence of CI-aCMBs, especially without typical signs of vasogenic edema on T_2_-weighted imaging, points towards a distinctive pathophysiological mechanism involving microvascular or capillary endothelial failure.

## Author contributions

**Conceptualization:** Bahadar S. Srichawla, Ton Fang.

**Data curation:** Bahadar S. Srichawla.

**Formal analysis:** Bahadar S. Srichawla.

**Funding acquisition:** Bahadar S. Srichawla.

**Investigation:** Bahadar S. Srichawla.

**Methodology:** Bahadar S. Srichawla, Rakhee Lalla.

**Project administration:** Bahadar S. Srichawla.

**Resources:** Bahadar S. Srichawla, Rakhee Lalla.

**Software:** Bahadar S. Srichawla.

**Supervision:** Bahadar S. Srichawla, Vincent Kipkorir, Rakhee Lalla.

**Validation:** Bahadar S. Srichawla, Rakhee Lalla.

**Visualization:** Bahadar S. Srichawla.

**Writing – original draft:** Bahadar S. Srichawla.

**Writing – review & editing:** Bahadar S. Srichawla.
